# Supplementary material for: Heat adaptation of phage T7 under an extended genetic code
Source: Virus Evol. 2021 Dec 1;7(2):veab100. doi: 10.1093/ve/veab100 (PMC8923235; doi:10.1093/ve/veab100)
Supplement: veab100_Supp [file veab100_supp.zip › Sequencing_Analysis.zip!/Sequencing_Analysis/A2/evidence/JC_SIDE_1_49.html]

BRESEQ :: Evidence


| New junction evidence | | | | | | | | | | | |
| --- | --- | --- | --- | --- | --- | --- | --- | --- | --- | --- | --- |
|  | | seq id | position | reads (cov) | reads (cov) | score | skew | freq | annotation | gene | product |
| \* | ? | NC\_001604.1 | = 13268 | 206 (0.450) | 5 (0.010) | 4/588 | 22.6 | 4.7% | intergenic (–/–) | *–/–* | –/– |
|  | ? | NC\_001604.1 | 14300 = | 1 (0.000) |  |  |  |  | intergenic (–/–) | *–/–* | –/– |
| Rejected: Coverage evenness skew score above cutoff. | | | | | | | | | | | |
| Rejected: Frequency below/above cutoff threshold. | | | | | | | | | | | |

Only 100 of 576 total aligned reads displayed.  

`CCCAGACAAAGGTAAAGCACATGAGGAAGGTCGCCCCGTTTCTATTACTGACCTACGTGGTTCTGGCGCACTACGCCAACTATCTGATACTATTATTGCCCTTGAGCGTAATCAGCAAGGCGATATGCCTAACCTTGTCCTCGTTCGTATTCTCAAGTGCCGCTTTACTGGTGATACTGGTATCGCTGGCTACATGGAATACAACAAGGAAACCGGATGGCTTGAACCATCAAGTTACTCAGGGGAAGAAGAGTCACACTCAGAGTCAACAGACTGGTCCAACGACACTGACTTCTGACAGGATTCTTGATGACTTTCCAGACGACTACGAGAAGTTTCGCTGGAGAGTCCCATTCTAATACGACTCACTAAAGGAGACACACCATGTTCAAACTGATTAAGAAGTTAGGCCAACTGCTGGTTCGTATGTACAACGTGGAAGCCAAGCGACTGAACGATGAGGCTCGTAAAGAGGCCACACAGTCACGCGCTCTGGCGATTCGCTCCAACGAACTGGCTGACAGTGCATCCACTAAAGTTACCGAGGCTGCCCGTGTGGCAAACCAAGCTCAACAGCTTTCCAAATTCTTTGAGTAATCA  >  NC_001604.1/12968‑13567`  

                                                                                                                                                                                                                                                                                                            `|`                                                                                                                                                                                                                                                                                                             
          `ggTAAAGCACATGAGGAAGGTCGCCCAGTTTCTATTACTGACCTACGTGGTTCTGGCGCACTACGCCAACTATCTGATACTATTATTGCCCTTGAGCGTAATCAGCAAGGCGATATGCCTAACCTTGTCCTAGTTCGTATTCTCAAGTGACGCTTTACTGGTGATACTGGTATCGCTGGCTACATGGAATACAACAAGGAAACCGGATGGCTTGAACCATCAAGTTACTCAGGGGAAGAAGAGTCACACTCAGAGTCAACAGACTGGTCCAACGACACTGACTTCTGACAGGATTCTtgat                                                                                                                                                                                                                                                                                                   >  1:648411/1‑301 (MQ=255)`  
                  `acaTGAGGAAGGTCGCCCCTTTTCTATTACTGACCTACGTGGTTCTGGCGCACTACGCCAACTATCTGATACTATTATTGCCCTTGAGCGTAATCAGCAAGGCGATATGCCTAACCTTGTCCTCGTTCGTATTCTCAAGTGCCGCTTTACTGGTGATACTGGTATCGCTGGCTACATGGAATACAACAAGGAAACCGGATGGCTTGAACCATCAAGTTACTCAGGGGAAGAAGAGTCACACTCAGAGTCAACAGACTGGTCCAACGACACTGACTTCTGACAGGATTCTTGAcagttgttt                                                                                                                                                                                                                                                                                           <  1:605575‑M1/301‑10 (MQ=255)`  
                     `tGAGGAAGGTCGCCCCGTTTCTATTACTGACCTACGTGGTTCTGGCGCACTACGCCAACTATCTGATACTATTATTGCCCTTGAGCGTAATCAGCAAGGCGATATGCCTAACCTTGTCCTCGTTCGTATTCTCAAGTGCCGCTTTACTGGTGATACTGGTATCGCTGGCTACATGGAATACAACAAGGAAACCGGATGGCTTGAACCATCAAGTTACTCAGGGGAAGAAGAGTCACACTCAGAGTCAACAGACTGGTCCAACGACACTGACTTCTGACAGGATTCTTGATGACTTTCCAga                                                                                                                                                                                                                                                                                        >  1:584045/1‑301 (MQ=255)`  
                     `tGAGGAAGGTCGACCCGTTTCTATTACTGACCTACGTGGTTCTGGCGCAATACGCCAACTATCTGATACTATTATTGCCCTTGAGCGTAATCAGCAAGGCGATATGCCTAACCTTGTCCTCGTTCGTATTCTCAAGTACCGCTTTACTGGTGATACTGGTATCGCTGGCTACATGGAATACAACAAGGAAACCGGATGGCTTGAACCATCAAGTTACTCAGGGGAAGAAGAGTCACACTCAGAGTCAACAGACTGGTCCAACGACACTGACTTCTGACAGGATTCTTGATGACTTTCCata                                                                                                                                                                                                                                                                                        <  1:693643/301‑3 (MQ=255)`  
                       `aGGAAGGTCTCCCCGTTTCTATTACTGACCTACGTGGTTCTGGCGCACTACGCAAACTATCTGATACTATTATTGCCGTTGAGCGTAATCAGCAAGGCGATATGCCTAACATTGTCCTCGTTCGTATTCTCAAGTGCCGCTTTACTGGTGATACTGGTATCGCTGGCTACATGGAATACAACAAGGAAACCGGATGGCTTGAACCATCAAGTTACTCAGGGGAAGAAGAGTCACACTCAGAGTCAACAGACTGGTCAAACGACACTGACTTCTGACAGGATTCTTGATTACTTTCCAgacg                                                                                                                                                                                                                                                                                      >  1:439093/1‑301 (MQ=255)`  
                             `tccgcCCCGTTTCTATTACTGACCTACGTGGTTCTGGCGCACTACGCCAACTATCTGATACTATTATTGCCCTTGAGCGTAATCAGCAAGGCGATATGCCTAACCTTGTCCTCGTTCGTATTCTCAAGTGCCGCTTTACTGGTGATACTGGTATCGCTGGCTATATGGAATACAACAAGGAAACCGGATGGCTTGAACCATCAAGTTACTCAGGGGAAGAAGAGTCACACTCAGAGTCAACAGACTGGTCCAACGACACTGACTTCTGACAGGTTTCTTGAcagttgtttcatatgaagag                                                                                                                                                                                                                                                                                <  1:318495‑M1/299‑21 (MQ=255)`  
                                      `tttCTATTACTGACCTACGTGGTTCTGGCGCACTACGCCAACTATCTGATACTATTATTGCCCTTGAGCGTAATCAGCAAGGCGATCTGCCTAACCTTGTCCTCGTTCGTATTCTCACGTGCCGCTTTACTGGTGATACTTGTATCGCTGGCTACATGGAATACAACAAGGAAACCTGATGGCTTGAACCATCAAGTTACTCAGTGGAAGAAGAGTCACACTCAGAGTCAACATACTGGTCCAACGACACTGACTTCTGACAGGATTCTTGAcagttgtttcatatgaagagattgttaag                                                                                                                                                                                                                                                                       <  1:134115‑M1/301‑30 (MQ=255)`  
                                      `tttCTATTACTGACCTACGTGGTTCTGGCGCACTACGCCAACTATCTGATACTATTATTGCCCTTGAGCGTAATCAGCAAGGCGATATGCCTAACCTTGTCCTCGTTCGTATTCTCAAGTGCCGCTTTACTGGTGATACTGGTATCGCTGGCTACATGGAATACAACAAGGAAACCGGATGGCTTGAACCATCAAGTTACTCAGGGGAAGAAGAGTCACACTCAGAGTCAACAGACTGGTCCAACGACACTGACTTCTGACAGGATTCTTGAcagttgtttcatatgaagagattgttaag                                                                                                                                                                                                                                                                       >  1:393359‑M1/1‑272 (MQ=255)`  
                                       `ttCTATTACTGACCTACGTGGTTCTGGCGCACTACGCCAACTATCTGATACTATTATTGCCCTTGAGCGTAATCAGCAAGGCGATATGCCTAACCTTGTCCTCGTTCGTATTCTCAAGTGCCGCTTTACTGGTGATACTGGTATCGCTGGCTACATGGAATACAACAAGGAAACCGGATGGCTTGAACCATCAAGTTACTCAGGGGAAGAAGAGTCACACTCAGAGTCAACAGACTGGTCCAACGACACTGACTTCTGACAGGATTCTTGAcagttgtttcatatgaagagattgttaagt                                                                                                                                                                                                                                                                      >  1:377334‑M1/1‑271 (MQ=255)`  
                                           `aTTACTGACCTACGTGGTTCTGGCGCACTACGCCAACTATCTGATACTATTATTGCCCTTGAGCGTAATCAGCAAGGCGATATGCCTAACCTTGTCCTCGTTCGTATTCTCAAGTGCCGCTTTACTGGTGATACTGGTATCGCTGGCTACATGGAATACAACAAGGAAACCGGATGGCTTGAACCATCAAGTTACTCAGGGGAAGAAGAGTCACACTCAGAGTCAACAGACTGGTCCAACGACACTGACTTCTGACAGGATTCTTGAcagttgtttcatatgaagagattgttaagtcacg                                                                                                                                                                                                                                                                  >  1:414199‑M1/1‑267 (MQ=255)`  
                                              `aCTTACCTACGTGGTTCTGGCGCACTACGCCAACTATCTGATACTATTATTGCCCTTGAGCGTAATCAGCAAGGCGATATGCCTAACCTTGTCCTCGTTCGTATTCTCAAGTGCCGCTTTACTGGTGATACTGGTATCGCTGGCTACATGGAATACAACAAGGAAACCGGATGGCTTGAACCATCAAGTTACTCAGGGGAAGAAGAGTCACACTCAGAGTCAACAGACTGGTCCAACGACACTGACTTATGACAGGATTCTTGAcagttgtttcatatgaagagattgttaagtcacgata                                                                                                                                                                                                                                                               <  1:46388‑M1/301‑38 (MQ=255)`  
                                                 `gACCTACGTGGTTCTGGCGCACTACGCCAACTATCTGATACTATTATTGCCCTTGAGCGTAATCAGCAAGGCGATATGCCTAACCTTGTCCTCGTTCGTATTCTCAAGTGCCGCTTTACTGGTGATACTGGTATCGCTGGCTACATGGAATACAACAAGGAAACCGGATGGCTTGAACCATCAAGTTACTCAGGGGAAGAAGAGTCACACTCAGAGTCAACAGATTGGTCCAACGACACTGACTT‑‑‑‑‑‑‑‑‑‑‑‑TTGAcagttgtttcatattaagagattgtttagtcacgataatcaataggagaaat                                                                                                                                                                                                                                                >  1:292755‑M1/1‑249 (MQ=255)`  
                                                       `ctcggagatgtgtataagagacagcTATCTGATACTATTATTGCCCTTGAGCGTAATCAGCAAGGCGATATGCCTAACCTTGTCCTCGTTCGTATTCTCAAGTGCCGCTTTACTGGTGATACTGGTATCGCTGGCTACATGGAATACAACAAGGAAACCGGATGGCTTGAACCATCAAGTTACTAAGGGGAAGAAGAGTCACACTCAGAGTCAACAGACTGGTCCAACGACACTGACTTATGACAGGATTCTTGATGACTTTCCAGACGACTACGAGAAGTTTCGCTGGAGAGTCCCATTc                                                                                                                                                                                                                                                      <  1:704304/277‑1 (MQ=255)`  
                                                           `gTTCTGGCGCACTACGCCAACTATCTGATACTATTATTGCCCTTGAGCGTAATCAGCAAGGCGATATGCCTAACTTTGTCCTCGTTCGTATTCTCAAGTGCCGCTTTACTGGTGATACTGGTATCGCTGGCTACATGGAATACAACTAGGAAACCGGATGGCTTGAACCATCAAGTTACTCAGGGGAAGAAGAGTCACACTCAGAGTTAACAGACTGGTCCAACGACACTGACTTCTGACAGGATTCTTGATGACTTTCCAGACGACTACGAGAAGTTTCGCTGGAGAGTCCCATTCTAAt                                                                                                                                                                                                                                                  >  1:219460/1‑301 (MQ=255)`  
                                                             `gaggtttgtataagagacagaTCTGATACTATTAGTGCGCTTGAGCGTAATCAGCAAGGCGATATGCCTAACCTTGTCCTCGTTCGTATTCTCAAGTGCCGCTTTACTGGTGATACTGGTATCGCTGGCTACATGGAATACAACAAGGAAACCGGATGGCTTGAACCATCAAGTTACTCAGGGGAAGAAGAGTCACACTCAGAGTCAACAGACTGGTCCAACGACACTGACTTCTGACAGGATTCTTGATGACTTTCCAGACGACTACGAGAAGTTTTGCTGGAGAGTCCCATTCTAATAc                                                                                                                                                                                                                                                <  1:22992/281‑1 (MQ=255)`  
                                                              `cTGGTGCACTACGCCAACTATCTGATACTATTATTGCCCTTGAGCGTAATCAGCAAGGCGATATGCCTAACCTTGTCCTCGTTCGTATTCTCAAGTGCCGCTTTACTGGTGATACTGGTATCGCTGGCTACATGGAATACAACAAGGAAACCGGATGGCTTTAACCATCAAGTTACTCAGGGGAAGAAGAGTCACACTCAGAGTCAACAGACTGGTCCAACGACACTGACTTCTGACAGGATTCTTGATGACTTTCCAGACGACTACGAGAAGTTTCGCTGGATAGTCCCATTCTAATACg                                                                                                                                                                                                                                               >  1:330089/1‑301 (MQ=255)`  
                                                              `cTGGCGCACTACGCCAACTATCTGATACTATTATTGCCCTTGAGCGTAATCAGCAAGGCGATATGCCTAACCTTGTCCTCGTTCGTATTCTCAAGTGCCGCTTTACTGGTGATACTGGTATCGCTGGCTACATGGAATACAACAATGAAACCGGATGGCTTGAACCATCAAGTTACTCAGGGGAAGAAGAGTCACACTCAGAGTCAACAGACTGGTCCAACTACACTGACTTCTGACAGGATTCTTGATGACTTTCCAGACGACTACTAGAAGTTTCGCTGGAGAGTCCCATTCTAATACg                                                                                                                                                                                                                                               >  1:157878/1‑301 (MQ=255)`  
                                                                `ggCGTACTACGCCAACTATCTGATACTATTATTGCCCTTGAGCGTAATCAGCAAGGCGATATGCCTAACCTTGTCCTCGTTCGTATTCTCAAGTGCCGCTTTACTGGTGATACTGGTATCGCTGGCTACATGGAATACAACAAGGAAACCGGATGGCTTGAACCATCAAGTTACTCAGGGGAAGAAGAGTCACACTCAGAGTCAACAGACTGGTCCAACGACACTGACTTCTGACAGGATTCTTGATGACTTTCCAGACGACTACGAGAAGTTTCGcctgtctcttatacacatctccgag                                                                                                                                                                                                                                             >  1:231381/1‑277 (MQ=255)`  
                                                                      `cTACGCCAACTATCTGATACTATTATTTCCCTTGAGCGTAATCAGCAAGGCGATATGCCTAACCTTGTCCTCGTTCGTATTCTCAAGTGCCGCTTTACTGGTGATACTGGTATCGCTGGCTACATGGAATACAACAAGGAAACCGGATGGCTTGAACCATCAAGTTACTCAGTGGAAGAAGAGTCACACTCAGAGTCAACAGACTGGTCCAACGACACTGACTTCTGACAGGATTCTTGATGACTTTCCAGACGACTACGAGAAGTTTCGCTGGAGAGTCCCATTCTAATACGactaacta                                                                                                                                                                                                                                       >  1:148614/1‑301 (MQ=255)`  
                                                                      `cTACGCCAACTATCTGATACTATTATTGCCCTTGAGCGTAATCAGCAAGGCGATATTCCTAACCTTGTCCTCGTTCGTATTCTCAAGTGCCGCTTTACTGGTGATACTGGTATCGCTGGCTACATGGAATACAACAAGGAAACCGGATGGCTTGAACCATCAAGTTACTCAGGGGAAGAAGAGTCACACTCAGAGTCAACAGACTGGTCCAACGACACTGACTTCTGACAGGATTCTTGATGACTTTCCAGACGACTACGAGAAGTTTCGCTGGAGAGTCCCATTCTAATACGACTCACTa                                                                                                                                                                                                                                       >  1:502996/1‑301 (MQ=255)`  
                                                                      `cTACGCCAACTATCTGATACTATTATTGCCCTTGAGCGTAATCAGCAAGGCGATATGCCTAACCTTGTCCTCGTTCGTATTCTCAAGTGCCGCTTTACTGGTGATACTGGTATCGCTGGCTACATGGAATACAACAAGGAAACCGGATGGCTTGAACCATCAAGTTACTCAGGGGAAGAAGAGTCACACTCAGAGTCAACAGACTGGTCCAATGACACTGACTTCTGACAGGATTCTTGATGACTTTCCAGACGACTACGAGAAGTTTCGCTGGAGAGTCCCATTcctgtctcttatacac                                                                                                                                                                                                                                       >  1:49274/1‑286 (MQ=255)`  
                                                                        `aCGCCAACTATCTGATACTATTATTGCCCTTGAGCGTAATCAGCAAGGCGATATGCCTAACCTTGTCCTCGTTCGTATTCTCAAGTGCCGCTTTACTGGTGATACTGGTATCGCTGGCTACATGGAATACAACAAGGCAACCGGATGGCTTGAACCATCAAGTTACTCAGGGGAAGAAGAGTCACACTCAGAGTCAACAGACTGGTCCAACGACACTGACTTCTGACAGGATTCTTGATGACTTTCCAGACTACTACGAGAAGTTTCGCATGAGAGTCACATTCTAATAAGACTAACTaaa                                                                                                                                                                                                                                     >  1:333840/1‑301 (MQ=255)`  
                                                                          `gCCAACTATCTGATACTATTATTGCCCTTGAGCGTAATCAGCAAGGCGATATGCCTAACCTGGTCCTCGTTCGTATTCTCAAGTGCCGCTTTACTGGTGATACTGGTATCGCTGGCTACATGGAATACAACAAGGAAACCGGATGGCTTGAACCATCAAGTTACTCAGGGGAAGAATAGTCACACTCAGAGTCAACAGACTGGTCCAACGACACTGACTTCTGACAGGATTCTTGATGACTTTCCAGACGACTACGAGAAGTTTCGCTGGAGAGTCCCATTCTAATACGACTCACTAAAgg                                                                                                                                                                                                                                   <  1:451395/301‑1 (MQ=255)`  
                                                                            `gtgtataagagacagattatttcccttgagcgtaatCAGCAAGGCGATATGCCTAACCTTGTCCTCGTTCGTATTCTCAAGTGCCGCTTTACTGGTGATACTGGTATCGCTGGCTACATGGAATACAACAAGGAAACCGGATGGCTTGAACCATCAAGTTACTCAGGGGAAGAAGAGTCACACTCAGAGTCAACAGACTGGTCCAACGACACTGACTTCTGACAGGTTTCTTGAcagttgtttcatatgaagagattgttaagtcacgataatcaataggagaaatcaatatgatcgtttc                                                                                                                                                                                                                                 <  1:115183‑M1/286‑68 (MQ=255)`  
                                                                                 `aTCTGATACTATTATTGCCCTTGAGCGTAATCAGCAAGGCGATATGCCTAACCTTGTCCTCGTTCGTATTCTCAAGTGCCGCTTTACTGGTGATACTGGTATCGCTGGCTACATGGAATACAACAAGGAAACCGGATGGCTTGAACCATCAAGTTACTCAGGGGAAGAAGAGTCACACTCAGAGTCAACAGACTGGTCCAACGACACTGACTTCTGACAGGATTCTTGAcagttgtttcatatgaagagattgttaagtcacgataatcaataggagaaatcaatatgatcgtttctgaca                                                                                                                                                                                                                            >  1:153539‑M1/1‑229 (MQ=255)`  
                                                                                  `tCTGATACTATTATTGCCCTTGAGCGTAATCAGCAAGGCGATATGCCTAACCTTGTCCTCGTTCGTATTCTCAAGTGCCGCTTTACTGGTGATACTGGTATCGCTGGCTACATGGAATACAACAAGGAAACCGGATGGCTTGAACCATCAAGTTACTCAGGGGAAGAAGAGTCACACTCAGAGTCAACAGACTGGTCCAACGACACTGACTTCTGACAGGATTCTTGATGACTTTCAAGACGACTACGAAAAGTTTCGCTGGAGAGTCCAATTCTAATACGACTCACTAAAGGAGacacac                                                                                                                                                                                                                           <  1:788963/301‑1 (MQ=255)`  
                                                                                         `cTATTATTGCCCTTGAGCGTAATCAGCAAGGCGATATGCCTAACCTTGTCCTCGTTCGTATTCTCAAGTGCCGCTTTACTGGTGATACTGGTATCGCTGGCTACATGGAATACAACAAGGAAACCGGATTGCTTGAACCATCTAGTTACTCAGGGGTAGACGATTCACACCCCGAGTCAACAGACTGGTCCAACGACACTGAC‑‑‑‑‑‑‑‑‑‑‑TTCTT‑Acagttgtttcatatgaatatattgttaagtcacgataatcaataggagaaatcaatatgatcgtttctgacatcgaagctaacgccctctta                                                                                                                                                                                                        >  1:48179‑M1/1‑209 (MQ=255)`  
                                                                                         `cTATTATTGCCCTTGAGCGTAATCAGCAAGGCGATATGCCTAACCTTGTCCTCGTTCGTATTCTCAAGTGCCGCTTTACTGGTGATACTGGTATCGCTGGCTACATGGAATAAAACAAGGAAACCGGATGGCTTGAACCATCAAGTTACTCAGGGGAAGAAGAGTCACACTCAGAGTCAACAGACTGGTCCAACGACACTGACTTCTGACAGGATTCTTGATGACTTTCCAGACGACTACGAGAAGTTTCGCTGGAGAGTCCCATTCTAATACAACTCACTAAAGGAGacctgtctcttat                                                                                                                                                                                                                    >  1:163928/1‑290 (MQ=255)`  
                                                                                         `cTATTATTGCCCTTGAGCGTAATCAGCAAGGCGATATGCCTAACCTTGTCCTCGTTCGTATTCTCAAGTGCCGCTGTACTGGTGATACTGGTATCGCTGGCTACATTGAATACAACAAGGAAACCGGATGGCTTGAACCATCAAGTTACTCATGGGAAGAAGAGTCACACTCAGAGTCAACAGACTGGTCCAACGACACTGACTTCTGACAGGATTCTTGATGACTTTCCAGACGACTACGAGAAGTTTCGCTGGAGAGTCCCATTCTAATACGACTCACTAAAGGAGACACACCATGTTc                                                                                                                                                                                                                    <  1:569285/301‑1 (MQ=255)`  
                                                                                             `tattGGCCTTGAGCGTAAGCAGCAAGGCGATATGCCTAACCTTGTCCTAGTTCGTATGCTCAAGTGCCGCTTTACTGGTGATACTGGTATCGCTGGCTACATGGAATACAACAAGGAAACCGGATGGCTTGAACCATCAAGGTACTCAGGGGAAGAAGAGTCACACTCAGAGTAAACAGACTGGTCCAACGACACTGACTTCTGACAGGATTCTTGATGACTTTCCAGACGACTACGAGAAGTTTCGCTGGAGAGTCCCATTCTAATACGACTCACTAAAGGAGACACACCATGTTCAAAc                                                                                                                                                                                                                <  1:743802/301‑1 (MQ=255)`  
                                                                                                 `gCCCTTGAGCGTAATCAGCAAGGCGATATGCCTAACCTTGTCCTCGTTCGTATTCTCAAGTGCCGCTTTACTGGTGATACTGGTATCGCTGGCTACATGGAATACAACAAGGAAACCGGATGGCTTGAACCATCAAGTTACTCAGTGGAAGAAGAGTCACACTCAGAGTCAACAGACTGGTCCAACGACACTGACTTCTGACAGGATTCTTGAcagttgtttcatatgaagagattgttaagtcacgataatcaataggagaaatcaatatgatcgtttctgacatcgaagctaacgacct                                                                                                                                                                                                            >  1:816046‑M1/1‑213 (MQ=255)`  
                                                                                                       `gAGCGTAATCAGCAAGGCGATATGCCTAACCTTGTCCTCGTTCGTATTCTCAAGTGCCGCTTTACTGGTGATACTGGTATCGCTGGCTACATGGAATACAACAAGGACACCGGATGGCTTGAACCATCAAGTTACTCAGGGGAAGAAGAGTCACACTCAGAGTCAACAGACTGGTCCAACGACACTGACTTCTGACAGGATTCTTGATGAATTTCCAGACGACTACGAGAAGTTTCGCTGGAGAGTCCCATTCTAATCCGACTCACTAAAGGAGACACACCATGTTCAAACTGATTaagaa                                                                                                                                                                                                      >  1:269974/1‑301 (MQ=255)`  
                                                                                                       `gAGCGTAATCAGCAAGGCGATATGCCTAACCTTGTCCTCGTTCGTATTCTAAAGTGCCGCTTTACTGGTGATACTGGTATCGCTGGCTACATGGAATACAACAAGGAAACCGGATGGCTTGAACCATCAAGTTACTCAGGGGAAGAAGAGTCACACTCAGAGTCAACAGACTGGTCCAACGACACTGACTTCTGACAGGATTCTTGATGACTTTCCAGACGACTACGAGAAGTTTCGCTGGAGAGTCCCATTCTAATACGACTCACTAAAGGAGACACTCCATGTTCAAACTGATTaagaa                                                                                                                                                                                                      >  1:749685/1‑301 (MQ=255)`  
                                                                                                        `ggaaatgtgtataagagtaattgcctatccttgtcctcgttcaTATTCTCAAGTGCCGCTTTACTGGTGATACTGGTATCGCTGGCTACATGGAATACAACAAGGAAACCGGATGGCTTGAACCATCAAGTTACTCAGGGGAAGAAGAGTCACACTCAGAGTCAACAGACTGGTCCAACGACACTGACTTCTGACAGGATTCTTGAcagttgtttcatatgaagagattgttaagtcacgataatcaataggagaaatcaatatgatcgtttctgacatcgaagctaacgccctcttagag                                                                                                                                                                                                     <  1:514411‑M1/280‑96 (MQ=255)`  
                                                                                                        `gacagaatcagcAAGGCGATATGCCTAACCTTGTCCTCGTTCGTATTCTCAAGTGCCGCTTTACTGGTGATACTGGTATCGCTGGCTACATGGAATACAACAAGGAAACCGGATGGCTTGAACCATCAAGTTACTCAGGGGAAGAAGAGTCACACTCAGAGTCAACAGACTGGTCCAACGACACTGACTTCTGACAGGATTCTTGAcagttgtttcatatgaagagattgttaagtcacgataatcaataggagaaatcaatatgatcgtttctgacatcgaagctaacgccctcttagag                                                                                                                                                                                                     <  1:78181‑M1/296‑96 (MQ=255)`  
                                                                                                        `cggagatgtgtataagagacaggCCTAACCTTGTCCTCGTTCGTATTCTCAAGTGCCGCTTTACTGGTGATACTGGTATCGCTGGCTACATGGAATACAACAAGGAAACCGGATGGCTTGAACCATCAAGTTACTCAGGGGAAGAAGAGTCACACTCAGAGTCAACAGACTGGTCAAACGACACTGACTTCTGACAGGATTCTTGATGACTTTCCAGACGACTACGAGAAGTTTCGCTGGAGAGTCCCATTCTAATACGACTCACTAAAGGAGACACACCATGTTCAAACTGATTaagaag                                                                                                                                                                                                     <  1:456418/279‑1 (MQ=255)`  
                                                                                                        `aGCGTAATCAGCAAGGTGATATGCCTAACCTTGTCCTCGTTCGTATTCTCAAGTGCCGCTTTACTGGTGATACTGGTATCGCTGGCTACATGGAATACAACAAGGAAACCGGATGGCTTGAACCATCAAGTTACTCAGGGGAAGAAGAGTCACACTCAGAGTCAACAGACTGGTCCAACGACACTGACTTCTGACAGGATTCTTGAcagttgtttcatatgaagagattgttaagtcacgataatcaataggagaaatcaatatgatcgtttctgacatcgaagctaacgcccctgtctct                                                                                                                                                                                                     >  1:276507‑M1/1‑206 (MQ=255)`  
                                                                                                        `aGCGTAATCAGCAAGGCGATATGCCTAACCTTGTCCTCGTTCGTATTCTCAAGTGCCGCTTTACTGGTGATACTGGTATCGCTGGCTACATGGAATACAACAAGGAAACCGGATGGCTTGAACCATCAAGTTACTCAGGGGAAGAAGAGTCACACTCAGAGTCAACAGACTGGTCCAACGACACTGACTTCTGACAGGATTCTTGAcagttgtttcatatgaagagattgttaagtcacgataatcaataggagaaatcaatatgatcgtttctgacatcgaagctaacgccctcttagag                                                                                                                                                                                                     <  1:23827‑M1/301‑96 (MQ=255)`  
                                                                                                        `aGCGTAATCAGCAAGGCGATATGCCTAACCTTGTCCTCGTTCGTATTCTCAAGTGCCGCTTTACTGGTGATACTGGTATCGCTGGCTACATGGAATACAACAAGGAAACCGGATGGCTTGAACCATCAAGTTACTCAGGGGAAGAAGAGTCACACTCAGAGTCAACAGACTGGTCCAACGACACTGACTTCTGACAGGATTCTTGATGACTTTCCAGACGACTACGAGACGTTTCGCTGGAGAGTCCCATTCTAATACGACTCACTAAAGGAGACACACCATGTTCAAACTGATTaagaag                                                                                                                                                                                                     >  1:81014/1‑301 (MQ=255)`  
                                                                                                                `cAGCAAGGCGATATGCCTAACCTTGTCCTCGTTCGTATTCTCAAGTGCCGCTTTACTGGTGATACTGGTATCGCTGGCTACATGGAATACAACAAGGAAACCGGATGGCTTGAACCATCAAGTTACTCAGGGGAAGAAGAGTCACACTCAGAGTCAACAGACTGGTCCAACGACACTGACTTCTGACAGGATTCTTGAcagttgtttcatatgaagagattgttaagtcacgataatcaataggagaaatcaatatgatcgtttctgacatcgaagctaacgccctgtctcttatacacat                                                                                                                                                                                             >  1:166417‑M1/1‑198 (MQ=255)`  
                                                                                                                         `gATATGCCTAACCTTGTCCTCGTTCGTATTCTCAAGTGCCGCTTTACTGTTGATACTGGTATCGCTGGCTACATGGAATACAACAAGGAAACCGGATGGCTTGAACCATCAAGTTACTCAGGGGAAGAAGAGTCACACTCAGAGTCAACAGTCTGGTCCAACGACACTGACTTCTGACAGGATTCTTGATGACTTTCCAGACGACTACGAGAAGTTTCGCTGGAGATTCCCATTCTAATACGACGCACTAAAGGAGACACACCAAGTTCAAACTGATTAAGAAGTTAGGCCAAcctgtctc                                                                                                                                                                                    >  1:673016/1‑294 (MQ=255)`  
                                                                                                                          `cagtgccTAACCTTGTCCTCGTTCGTATTCTCAAGTGCCGCTTTACTTGTGATACTGGTATCGCTGGCTACATGGAATACAACAAGGAAACCGGATGGCTTGAACCATCAAGTTACTCATGGGAAGAAGAGTCACACTCAGAGTCAACAGACTGGTCCAACGACACTGACTTCTGACAGGATTCTTGAcagttgtttcatatgaagagattgttaagtcacgataatcaataggagaaatcaatatgatcgtttctgacatcgaagctaacgccctcttagagagcgtcactaagttccac                                                                                                                                                                                   >  1:542253‑M1/4‑188 (MQ=255)`  
                                                                                                                          `atatGCCTAACCTTGTCCTCGTTCGTATTCTCAAGTGCCGCTTTACTGGTGATACTGGTATCGCTGGCTACATGGAATACAACAAGGAAACCTGATGGCTTGAACCATCAAGTTACTCAGGGGAAGAAGAGTCACACTCAGAGTCAACAGACTGGTCCAACGACACTGACTTC‑‑‑‑‑‑‑‑‑‑‑‑TGAcagttgtttcatatgaagagattgttaagtcacgataatcaataggagaaatcaatatgatcgtttctgacatcgaagctaacgccctcttagagagcgtcactaagttccactgcggggttatc                                                                                                                                                                       <  1:175682‑M1/301‑126 (MQ=255)`  
                                                                                                                                `cTAACCTTGTCCTCGTTCGTATTCTCAAGTGCCGCTTTACTGGTGATACTGGTATCGCTGGCTACATGGAATACAACAAGGAAACCGGATGGCTTGAACCATCAAGTTACTCAGGGGAAGAAGAGTCACACTCAGAGTCAACAGACTGGTCCAACGACACTGACTTCTGACAGGATTCTTGATGACTTTCCAGACGACTACGAGAAGTTTCGCTGGAGAGTCCCATTCTAATACGACTCACTAAAGGAGACACACCATGTTCAAACTGATTAAGAAGTTAGGCCAACTGCTGGTTCATATg                                                                                                                                                                             <  1:683113/301‑1 (MQ=255)`  
                                                                                                                                  `aaCCTTGTCCTCGTTCGTATTCTCAAGTGCCGCTTTACTGGTGATACTGGTATCGCTGGCTACATGGAATACAACAAGGAAACCGGATGGCTTGAACCATCAAGTTACTCAGGGGAAGAAGAGTCACACTCAGAGTCAACAGACTGGTCCAACGACACTGACTTC‑‑‑‑‑‑‑‑‑‑‑‑TGAcagttgtttcatatgaagagattgttaagtcacgataatcaataggagaaatcaatatgatcgtttctgacatagaagctaacgccctcttagagagcgtcactaagttccctgtctcttatacacatctccg                                                                                                                                                               >  1:526363‑M1/1‑168 (MQ=255)`  
                                                                                                                                        `gTCCTCGTTCGTATTCTCAAGTGCCGCTTTACTGGTGATACTGGTATCGCTGGCTACATTGAATACAACAAGGAAACCGGATGGCTTGAACCATCAAGTTACTCAGGGGAAGAAGAGTCACACTCAGAGTCAACAGACTGGTCCAACGACACTGACTTCTGACAGGATTCTTGATGACTTTCCAGACGACTACGAGAAGTTTCGCTGGAGAGTCCCATTCTAATACGACTCACTAAAGGAGACACACCATGTTCAAACTGATTAAGAAGTTAGGCCAACTGCTTGTTCGTATGTACAACGt                                                                                                                                                                     >  1:724379/1‑301 (MQ=255)`  
                                                                                                                                            `ggctcggagatgtgtataagagacagactggtgatactggtatcgctggctacATGGAATACAACAAGGAAACCGGATGGCTTGAACCATCAAGTTACTCAGGGGAAGAAGAGTCACACTCAGAGTCAACAGACTGGTCCAACGACACTGACTTCTGACAGGATTCTTGAcagttgtttcatatgaagagattgttaagtcacgataatcaataggagaaatcaatatgatcgtttctgacatcgaagctaacgccctcttagagagcgtcactaagttccactgcggggttatctacgac                                                                                                                                                                 <  1:124372‑M1/275‑132 (MQ=255)`  
                                                                                                                                            `aggttcgtatTCTCAAGTGCCGCTTTACTGGTGATACTGGTATCGCTGGCTACATGGAATACAACAAGGAAACCGGATGGCTTGAACCATCAAGTTACTCAGGGGAAGAAGAGTCACACTCAGAGTCAACAGACTGGTCCAACGACACTGACTTCTGACAGGATTCTTGAcagttgtttcatatgaagagattgttaagtcacgataatcaataggagaaatcaatatgatcgtttctgacatcgaagctaacgccctcttagagagcgtcactaagttccactgcggggttatctacgac                                                                                                                                                                 <  1:360646‑M1/299‑132 (MQ=255)`  
                                                                                                                                             `cgttcgtATTCTCAAGTGCCGCTTTACTGGTGATACTGGTATCGCTGGCTACATGGAATACAACAAGGAAACCGGATGGCTTGAACCATCAAGTTACTCAGGGGAAGAAGAGTCACACTCAGAGTCAACAGACTGGTCCAACGACACTGACTTCTGACAGGATTCTTGATGACTTTCCAGACGACTACGAGAAGTTTCGCTGGAGAGTCCCATTCTAATACGACTCACTAAAGGAGACACACCATGTTCAAACTGATTAAGACGTTAGGCCAACTGCTGGTTCGTATGTACAACGTGGAAg                                                                                                                                                                >  1:388514/1‑301 (MQ=255)`  
                                                                                                                                                 `ggctcggagatgtgtataagagacagtgatactggtatcgctggctacatgGAATACAACAAGGAAACCGGATGGCTTGAACCATCAAGTTACTCAGGGGAAGAAGAGTCACACTCAGAGTCAACAGACTGGTCCAACGACACTGACTTCTGACAGGATTCTTGAcagttgtttcatatgaagagattgttaagtcacgataatcaataggagaaatcaatatgatcgtttctgacatcgaagctaacgccctcttagagagcgtcactaagttccactgcggggttatctacgactactc                                                                                                                                                            <  1:101125‑M1/276‑137 (MQ=255)`  
                                                                                                                                                      `tcgcAAGTGCCGCTTTACTGGTGATACTGGTATCGCTGGCTACATGGAATACAACAAGGAAACCAGATGGCTTGAACCATCAAGTTACTCAGGGGAAGAAGAGTCACACTCAGAGTCAACAGACTGGTCCAACGACACTGACTTCTGACAGGATTCTTGATGACTTTCCAGACGACTACGAGAAGTTTCGCTGGAGAGTCCAATTCTAATACGACTCACTAAAGGAGACACACCATGTTCAAACTGATTAAGAAGTTAGGCCAACTGCTGGTTCGTATGTACAACGTGGAAGCCAAGCGAc                                                                                                                                                       <  1:204456/298‑1 (MQ=255)`  
                                                                                                                                                       `tcggagatgtgtataagagaaagtactggtatcgctggctacatggaATACAACAAGGAAACCGGATGGCTTGAACCATCAAGTTACTCAGGGGAAGAAGAGTCACACTCAGAGTCAACAGACTGGTCCAACGACACTGACTTC‑‑‑‑‑‑‑‑‑‑‑‑TGAcagttgtttcatatgaagagattgttaagtcacgataatcaataggagaaatcaatatgatcgtttctgacatcgaagctaacgccctcttagagagcgtcactaagttccactgcggggttatctacgactactccaccgctgagtacgtaag                                                                                                                                          <  1:677385‑M1/278‑155 (MQ=255)`  
                                                                                                                                                       `ctcAAGTGCCGCTTTACTGGTGATACTGGTATCGCTGGCTACATGGAATACAACAAGGAAACCGGATGGCTTGAACCATCAAGTTACTCAGGGGAAGAAGAGTCACACTCAGAGTCAACAGACTGGTCCAACGACACTGACTTCTGACAGGATTCTTGAcagttgtttcatatgaagagattgttaagtcacgataatcaataggagaaatcaatatgatcgtttctgacatcgaagctaacgccctcttagagagcgtcactaagttccactgctgggttatctacgactactccaccgc                                                                                                                                                      >  1:588617‑M1/1‑159 (MQ=255)`  
                                                                                                                                                       `ctcAAGTGCCGCTTTACTGGTGATACTGGTATCGCTGGCTACATGGAATACAACAAGGAAACCGGATGGCTTGAACCATCAAGTTACTCAGGGGAAGAAGAGTCACACTCAGAGTCAACAGACTGGTCCAACGACACTGACTTCTGACAGGATTCTTGATGACTTTCCAGACGACTACGAGAAGTTTCGCTGGAGAGTCCCATTCTAATACGACTCACTAAAGGAGACACACCATGTTCAAACTGATTAAGAAGTTAGGCCAACTGCTGGTTCGTATGTACAACGTGGAAGcctgtctctt                                                                                                                                                      >  1:824272/1‑293 (MQ=255)`  
                                                                                                                                                         `aaagTGCCACTTTACTGGTGATACTGGTATCGCTGGCTACATGGAATACAACAAGGAAACCGGATGGCTTGAACCAGCAAGTGAGTCAGGGGAAGAAGAGTCACACTCAGAGTCAACAGACTGGTCCAACGACGCTGACGTCTGACAGGATTCTTGAcagttgtttcatatgaagagattgataagtcacgataatcaataggagaaatcaaaatgatcgtttctgacatcgaagctaacgccctcttagagagcgtcactaagttccactgcggggttatctacgactactccaccgctg                                                                                                                                                    <  1:211459‑M1/300‑145 (MQ=255)`  
                                                                                                                                                            `gTGCCGCTTTACTGGTGATACTGGTATCGCTGGCTACATGGAATACAACAAGGAAACCGGATGGCTTGAACCATCAAGTTACTCAGGTGAAGAAGAGTCACACTCAGAGTCAACAGACTGGTCCAACGACACTGACTTCTGACAGGATTCTTGATGACTTTCCAGACGACTACGAGAAGTTTCGCTGGAGAGTCCCATTCTAATACGACTCACTAAAGGAGACACACCATGTTCAAACTGATTAAGAAGTTAGGCCAACTGCTGGTTCGTATGTACAACGTGGAAGCCAAGCGACTTAACg                                                                                                                                                 >  1:366578/1‑301 (MQ=255)`  
                                                                                                                                                                 `gCTTTACTGGTGATACTGGTATCGCTGGCTACATTGAATACAACAAGGAAACCGGATGGCTTGAACCATCAAGTTACTCAGGGGAAGAAGAGTCACACTCAGAGTCAACAGACTGGTCCAACGACACTGACTTCTGACAGGATTCTTGATGACTTTCCAGACGACTACGAGAAGTTTCGCTGGAGAGTCCCATTCTAATACGACTCACTAAAGGAGACACACCATGTTCAAACTGATTAAGAAGTTAGGCCAACTGCTGGTTCGTATGTACAACGTGGAAGCCAAGCGACTGAACGATGAg                                                                                                                                            <  1:49661/301‑1 (MQ=255)`  
                                                                                                                                                                      `aCTGGTGATACTGGTATCGCTGGCTACATGGAATACAACAAGGAAACCGGATGGCTTGAACCATCAAGTTACTCAGGGGAAGAAGAGTCACACTCAGAGTCAACAGACTGGTCCAACGACACTGACTTATGACAGGATTCTTGATGACTTTCCAGACGACTACGAGAAGTTTCGCTGGAGAGTCCCATTCTAATACGACTCACTAAAGGAGACACACCATGTTCAAACTGATTAAGAAGTTAGGCCAACTGCTGGTTCGTATGTACAACGTGGAAGCCAAGCGACTGAACGATGAGGCTCg                                                                                                                                       >  1:508292/1‑301 (MQ=255)`  
                                                                                                                                                                       `cTGGTGATACTGGTATCGCTGGCTACATGGAATACAACAAGGAAACCGGATGGCTTGAACCATCAAGTTACTCAGGGGAAGAAGAGTCACACTCAGAGTCACCAGACTGGTCCAACGACACTGACTTCTGACAGGATTCTTGAcagttgtttcatatgaagagattgttaagtcacgataatcaataggagaaatcaatatgatcgtttctgacatcgaagctaacgccctcttagagagcgtcactaagttccactgcagggttatctacgcctactccaccgctgagtacgcacggtcc                                                                                                                                      >  1:425812‑M1/1‑143 (MQ=255)`  
                                                                                                                                                                       `ataagagacagggtatcgctggctACATGGAATACAACAAGGAAACCGGATGGCTTGAACCATCAAGTTACTCAGGGGAAGAAGAGTCACACTCAGAGTCAACAGACTGGTCCAACGACACTGACTTCTGACAGGATTCTTGAcagttgtttcatatgaagagattgttaagtcacgataatcaataggagaaatcaatatgatcgtttctgacatcgaagctaacgccctcttagagagcgtcactaagttccactgcggggttatctacgactactccaccgctgagtacgtaagctac                                                                                                                                      <  1:350194‑M1/290‑159 (MQ=255)`  
                                                                                                                                                                         `tgtctcgtagatgtgtataagagacaggaatacaacaagtaaaccggatggcttGAACCATCAAGTTACTCAGGGGAAGAAGAGTCACACTCAGCGTCAACAGACTGGTCCAACGACACTGACTTCTTACAGGTTTCTTGTcagttgtttcatatgaagagattgttaagtcacgataatcaataggagaaatcaatatgatcgtttctgacatcgaagctaacgccctcttagagagcgtcactaagttccactgcggggttatctacgactactccaccgctgagtacgtaagctaccg                                                                                                                                    <  1:344977‑M1/275‑161 (MQ=255)`  
                                                                                                                                                                         `gggctcggagatgggtataagaaacaggAATACAACAAGGAAACCGGATGGCTTGAACCATCAAGTTACTCAGGGGAAGAAGAGTCACACTCAGAGTCAACAGACTGGTCCAACGACACTGACGTCTGACAGGATTCTTGATGACTTTCCAGACGACTACGAGAAGTTTCGCTGGAGAGTCCCATTCTAATACGACTCACTAAAGGAGACACACCATGTTCAAACTGATTAAGAAGTTAGGCCAACTGCTGGTTCGTATGTACAACGTGGAAGCCAAGCGACTGAACGATGAGGCTCGTaa                                                                                                                                    <  1:559077/275‑1 (MQ=255)`  
                                                                                                                                                                         `ggTGATACTGGTATCGCTGGCTACATGGAATACAACAAGGAAACCGGATGGCTTGAACCATCAAGTTACTCAGGGGAAGAAGAGTCACACTCAGAGTCAACAGACTGTTCCAACGACACTGACTTCTGACAGGATTCTTGATGACTTTCCAGACGACTACGAGAAGTTTCGCTGGAGAGTCCCATTCTAATACGACTCACTAAAGGAGACACACCATGTTCAAACTGATTAAGAAGTTAGGCCAACTGCTGGTTCGTATGTACAACGTGGAAGCCAAGCGACTGAACGATGAGGCTCGTaa                                                                                                                                    >  1:94836/1‑301 (MQ=255)`  
                                                                                                                                                                         `ggTGATACTGGTATCGCTGGCTACATGGAATACAACAAGGAAACCGGATGGCTTGAACCATCAAGTTACTCAGGGGAAGAAGAGTCACACTCAGAGTCAACAGACTGGTCCAACGACACTGACTTCTGACAGGATTCTTGATGACTTTCCAGACGACTACGAGAAGTTTCGCTGGAGAGTCCCATTCTAATACGACTCACTAAAGGAGACACACCATGTTCAAACTGATTAAGAAGTTAGGCCAACTGCTGGTTCGTATGTACAACGTGGAAGCCAAGCGACTGAACGATGAGGCTCGTaa                                                                                                                                    <  1:516824/301‑1 (MQ=255)`  
                                                                                                                                                                         `ggTGATACTGGTATCGCTGGCTACATGGAATACAACAAGGAAACCGGATGGCTTGAACCATCAAGTTACTCAGGGGAAGAAGAGTCACACTCAGAGTCAACAGACTGGTCCAACGACACTGACTTCTGACAGGATTCTTAATGACTTTCCAGACGACTACGAGAAGTTTCGCTGGAGAGTCCCATTCTAATACGACTCACTAAAGGAGACACACCATGTTCAAACTGATTAAGAAGTTAGGCCAACTGCTGGTTCGTATGTACAACGTGGAAGccctgtctcttatacacatctccgatcc                                                                                                                                    >  1:632464/1‑275 (MQ=255)`  
                                                                                                                                                                         `ggTGATACTGGTATCGCTGGCTACATGGAATACAACAAGGAAACCGGATGGCTTGAACCAACAAGTTACTAAGGGGAAGAAGAGTCACACTCAGAGTCAACAGACTGGTCCAACGACACTGACTTCTGACAGGATTCTTGATGACTTTCCAGACGACTACGAGAAGTTTCGCTGGAGAGTCCCATTCTAATACGACTCACTAAAGGAGACACACCATGTTCAAACTGATTAAGAAGTTAGGCCAACTGCTGGTTCGTATGTACAACGTGGAAGCCAAGCGACTGAACGATGAGGCTCGTaa                                                                                                                                    <  1:718096/301‑1 (MQ=255)`  
                                                                                                                                                                           `tGATACTGGTATCGCTGGCTACATGGAATACAACAAGGAAACCGGATGGCTTGAACCATCAAGTTACTCAGGGGAAGAAGAGTCACACTCAGAGTCAACAGACTGGTCCAACGACACTGACTTCTGACAGGATTCTTGATGACTTTCCAGACGACTACGAGAAGTTTCGCTGGAGAGTCCCATTCTAATACGACTCACTAAAGGAGACACACCATGTTCAAACTGATTAAGAAGTTAGGCCAACTGCTGGTTCGTATGTACAACGTGGAAGCCAAGCGACTGAACGATGAGGCTCGTAAag                                                                                                                                  >  1:11082/1‑301 (MQ=255)`  
                                                                                                                                                                           `ggATACTGGTATCACTGGCTACATGGAATACAACAAGGAAACCGGATGGCTTGAACCATCAAGTTACTCAGGGGAAGAAGAGTCACACTCAGAGTCAACAGACTGGTCCAACGACACTGACTTCTGACAGGATTCTTGATGACTTTCCAGACGACTACAAGAAGTTTCGCTGGAGAGTCCCATTCTAATACGACTCACTAAAGGAGACACACCATGTTCAAACTGATTAAGAAGTTAGGCCAACTGCTGGTTCGTATGTACAACGTGGAAGCCAAGCGACTGAACGATGAGGCTCGTAAag                                                                                                                                  <  1:17937/300‑1 (MQ=255)`  
                                                                                                                                                                              `tACTGGTATCGCTGGCTACATGGAATACAACAAGGAAACCGGATGGCTTGAACCATCAAGTTACTCAGGGGAAGAAGAGTCACACTCAGAGTCAACAGACTGGTCCAACGACACTGACTTCTGACAGGATTCTTGATGACTTTCCAGACGACTACGAGAAGTTTCGCTGGAGAGTCCCATTCTAATACGACTCACTAAAGGAGACACACCATGTTCAAACTGATTAAGAAGTTAGGCCAACTGCTGGTTCGTATGTACAACGTGGAAGCCAAGCGACTGAACGATGAGGCTCGTAAAGAgg                                                                                                                               >  1:772139/1‑301 (MQ=255)`  
                                                                                                                                                                                  `ggTATCGCTGGCTACATGGAATACAACAAGGAAACCGGATGGCTTGAACCATCAAGTTACTCAGGGGAAGAAGAGTCACACTCATAGTCAACAGACTGGTCCAACGACACTGACTTCTGACAGGATTCTTGATGACTTTCCAGACGACTACGAGAAGTTTCGCTGGAGAGTCCCATTCTAATACGACTCACTAAAGGAGACACACCATGTTCAAACTGATTAAGAAGTTAGGCCAACTGCTGGTTCGTATGTACAACGTGGAAGCCAAGCGACTGAACGATGAGGCTCGTAAAGAGGCcac                                                                                                                           >  1:138852/1‑301 (MQ=255)`  
                                                                                                                                                                                   `gTATCGCTGGCTACATGGAATACAACAAGGAAACCGGATGGCTTGAACCATCAAGTTACTCAGGGGAAGAAGAGTCACACTCAGAGTCAACAGACTGGTCCAACGACACTGACTTCTGACAGGATTCTTGAcagttgtttcatatgaagagattgttaagtcacgataatcaataggagaaatcaatatgatcgtttctgacatcgaagctaacgccctcttagagagcgtcactaagttcaactgcggggttatctacgactactcaaccgctgagtacgtaagctaccgttcgagtgac                                                                                                                          >  1:802042‑M1/1‑131 (MQ=255)`  
                                                                                                                                                                                      `tCGCTGGCTACATGGAATACAACAAGGAAACCGGATGGCTTGAACCATCAAGTTACTCATGGGAAGAAGAGTCACACTCAGAGTCAACAGACTGGTCCAACGACACTGACTTCTGACAGGATTCTTGAcagttgtttcatatgaagagattgttaagtcacgataatcaataggagaaatcaatatgatcgtttctgacatcgaagctaacgccatcttagagagcgtcactaagttccactgcggggttatctacgactactccaccgctgagtacgtaagctaccgtccgagtgacttc                                                                                                                       >  1:752036‑M1/1‑128 (MQ=255)`  
                                                                                                                                                                                       `cGCTGGCTACATGGAATACAACAAGGAAACCGGATGGCTTGAACCATCAAGTTACTCAGGGTAAGAAGAGTCACACTCAGAGTCAACAGACTGGTCCAACGACACTGACTTCTGACAGGATTCTTGATGACTTTCCAGATGACTACGAGAAGTTTCGCTGGAGAGTCCCATTCTAATACGACTCACTAAAGGAGACACACCATGTTCAAACTGATTAAGAAGTTAGGCCAACTGCTGGTTCGTATGTACAACGTGGAAGCCAAGCGACTGAACGATGAGGCTCGTAAAGAGGCcacacctg                                                                                                                      >  1:191808/1‑298 (MQ=255)`  
                                                                                                                                                                                        `gCTGGCTACATGGAATACAACAAGGAAACCGGATGGCTTGAACCATCAAGTTACTCAGGGGAAGAAGAGTCACACTCAGAGTCAACAGACTGGTCCAACGACACTGACTTCTGACAGGATTCTTGATGACTTTCCAGACGACTACGAGAAGTTTCGCTGGAGAGTCCCATTCTAATACGACTCACTAAAGGAGACACACCATGTTCAAACTGATTAAGAAGTTAGGCCAACTGCTGGTTCGTATGTACAACGTGGAAGCAAAGCGACTGAACGATGAGGCTCGTAAAGAGGCCACACAGTC                                                                                                                     <  1:574630‑M1/301‑1 (MQ=255)`  
                                                                                                                                                                                          `agtgtacatGGAATACAAAAAGGAAATCGGATGTCTTTAACCATCAAGTTACTAAGGGGAAGAAGAGTCACACTCAGAGTCAACATACTGGTCCAACGACACTGACTTCTGACAGGATTCTTGATGACTTTCCAGACGACTACGAGAAGTTTCGCTGGAGAGTCCTATTCTAATACGACTCACTAAAGGAGACACACCATGTTCAAACTGATTAAGAAGTTAGGCCAACTGCTGGTTCGTATGTACAACGTGGAAGCCAAGCGACTGAACGATGAGGCTCGTAAAGAGGCCACACAGTCAC                                                                                                                   <  1:760596‑M1/297‑1 (MQ=255)`  
                                                                                                                                                                                               `aCATGGAATACAACAAGGAAACCGGATGGCTTGAACCATCAAGTTACTCAGGGGAAGAAGAGTCACACTCAGAGTCAACAGACTGGTCCAACGACACTGACTTCTGACAGGATTCTTGATGACTTTCCAGACGACTACGAGAAGTTTCGCTGGAGAGTCCCATTCTAATACGACTCACTAAAGGAGACACACCATGTTCAAACTGATTAAGAAGTTAGGCCAACTGCTGGTTAGTATGTACAACGTGGAAGCCAAGCGACTGAACGATGAGGCTCGTAAAGAGGCCACACAGTCACGataa                                                                                                              <  1:662244‑M1/301‑5 (MQ=255)`  
                                                                                                                                                                                                    `tcggagatgtgtataagagacaggcttgaaccatcaagttactcagGGGAAGAAGAGTCACACTCAGAGTCAACAGACTGGTCCAACGACACTGACTTCTGACAGGATTCTTGAcagttgtttcatatgaagagattgttaagtcacgataatcaataggagaaatcaatatgatcgtttctgacatcgaagctaacgccctcttagagagcgtcactaagttccactgcggggttatctacgactactccaccgctgagtacgtaagctaccgtccgagtgacttcggtgcgtatctgga                                                                                                         <  1:698827‑M1/279‑188 (MQ=255)`  
                                                                                                                                                                                                    `gAATACAACAAGGAAACCGGATGGCTTGAACCATCAAGTTACTCAGGGGAAGAAGAGTCACACCCAGAGTCAACAGACTGGTACAACGACACTGACTTCTGACAGGATTCTTGAcagttgtttcatatgaagagattgttaagtcacgataatcaataggagaaatcactatgatcgtttctgacatcgaagctaacgccctcttatagagcgtcactaagttccactgcggggttatctacgactactccaccgctgcgtacgtacgctcccgtccgagtgccttcggtgcgtatcttgt                                                                                                         >  1:41797‑M1/1‑114 (MQ=255)`  
                                                                                                                                                                                                       `aagagacaggaaaccGGATGGCTTGAACCATCAAGTTACTCAGGGGAAGAAGAGTCACACTCAGAGTCAACAGACTGGTCCAACGACACTGACTTCTGACAGGATTCTTGATGACTTTCCAGACGACTACGAGAAGTTTCGCTGGAGAGTCCCATTCTAATACGACTCACTAAAGGAGACACACCATGTTCAAACTGATTAAGAAGTTAGGCCAACTGCTGGTTCGTATGTACAACGTGGAAGCAAAGCGACTGAACGATGAGGCTCGTAAAGAGGCCACACAGTCACGataatcaatagg                                                                                                      <  1:622687‑M1/294‑13 (MQ=255)`  
                                                                                                                                                                                                           `gtgggctcggagatgtgtataagagacagagttactcaggggaagaagagtcacactcaGAGTCAACAGACTGGTCCAACGACACTGACTTCTGACAGGATTCTTGAcagttgtttcatattaagagattgttaagtcacgataatcaataggagaaatcaatatgatcgtttctgacatcgaagctaacgccctcttagagagcgtcactaagttccactgcggggttatctacgactactccaccgctgagtacgtaagctaccgtccgagtgacttcggtgcgtatctggatgcgctg                                                                                                  <  1:26457‑M1/272‑195 (MQ=255)`  
                                                                                                                                                                                                           `acaaGGAAACCGGATGGCTTGAACCATCAAGTTACTCAGGGGAAGAAGAGTCACACTCAGAGTCAACAGACTGGTCCAACGACACTGACTTCTGACAGGATTCTTGATGACTTTCCAGACGACTACGAGAAGTTTCGCTGGAGAGTCCCATTCTAATACGACTCACTAAAGGAGACACACCATGTTCAAACTGATTAAGAAGTTAGGCCAACTGCTGGTTCGTATGTACAACGTGGAAGCCAAGCGACTGAACGATGAGGCTCGTAAAGAGGCCACACAGTCACGataatcaataggagaa                                                                                                  >  1:24222‑M1/1‑285 (MQ=255)`  
                                                                                                                                                                                                                `gAAACCGGATGGATAGAACCATCAAGTTACTCAGGGGAAGAATAGCCACACTCAGAGTCAAAAGACTGGTCAAACGACAATAACTTATGACAGGATGCTTGAcagttgtttcatatgaagggattgttaagtcacgagaagaaataggagaaataaatatgatcgtttctgacatcgaagctaacgccctcttagagagcgtcactaagttccactgcggggttatctacgactactccaccgctgagtacgtaagctaccgtccgagtgacttcggtgcgtatctggatgcgctagaagc                                                                                             <  1:473790‑M1/301‑200 (MQ=255)`  
                                                                                                                                                                                                                      `ggATGGCTTTAACCATCAAGTTACTCAGGGGAAGAAGAGTCACCCTCAGAGTCAACTGACTGGTCCAACGACACTGACTTCTGACAGGATTCTTGATGGCTTTCCAGACGACTACGAGAAGTTTCGCTGGAGAGTCCCATTCTATTACGACGCACTAAAGGAGACACACCATGTTCAAACTGATTAAGAAGTTAGGCCAACTGGTGGTTCGTATGTACAGCGTGGAAGCCAAGCGACTGAACGATGAGGCTCGTAAAGAGGCCACACAGTCACGataatcaataggagaaatcaatatgat                                                                                       <  1:29996‑M1/301‑28 (MQ=255)`  
                                                                                                                                                                                                                                         `agagacagggggaagaaaagTCACACTCATAGTCAACAGACTGGTCCAACGACACTGACGTCTGACAGGATTCTTGATGACTTTCCAGACGACTACGAGAAGTTTCGCTGGAGAGTCCAATTCTAATACGACTCACTAAAGGAGACACACCATGTTCAAACTGATTAAGAAGTTAGGCCAACTGCTGGTTCGTATGTACAACGTGGAAGCCAAGCGACTGAACGATGAGGCTCGTAAAGAGGCCACACAGTCACGataatcaataggagaaatcaatatgatcgtttctgacatcgaagct                                                                    <  1:408236‑M1/293‑47 (MQ=255)`  
                                                                                                                                                                                                                                                 `ggggAAGAAGAGTCACACTCAGAGTCAACAGACTGGTCCAACGACACTGACTTCTGACAGGATTCTTGATGACTTTCCAGACGACTACGAGAAGTTTCGCTGGAGAGTCCCATTCTAATACGACTCACTAAAGGAGACACACCATGTTCAAACTGATTAAGAAGTTAGGCCAACTGCTGGTTCGTATGTACAACGTGGAAGCCAAGCGACTGAACGATGAGGCTCGTAAAGAGGCCACACAGTCACGataatcaataggagaaatcactatgatcgtttctgacatcgaagctaacgccct                                                            >  1:25070‑M1/1‑247 (MQ=255)`  
                                                                                                                                                                                                                                                  `gggAAGAAGAGTCACACTCAGAGTCAACAGACTGGTCAAACGACACTGACTTCTGACAGGATTCTTGAcagttgtttcatatgaagagattgttaagtcacgataatcaataggagaaatcaatatgatagtttctgacatcgaagctaacgccctcttagagagcgtcactaagttccactgcggggttatctacgactactccaccgctgagtacgtaagctaccgtccgagtgacttcggtgcgtatctggatgcgctggaagccgaggttgcacgaggcggtcttattgtgttccac                                                           <  1:732624‑M1/301‑234 (MQ=255)`  
                                                                                                                                                                                                                                                    `gaagaaGAGTCACACTCAGAGTCAACAGACTGGTCCAACGACACTGACTTCTGACAGGATTCTTGATGACTTTCCAGACGACTACGAGAAGTTTCGCTGGAGAGTCCCATTCTAATACGACTCACTAAAGGAGACACACCATGTTCAAACTGATTAAGAAGTTAGGCCAACTGCTGGTTCGTATGTACAACGTGGAAGCCAAGCGACTGAACGATGAGGCTCGTAAAGAGGCCACACAGTCACGataatcaataggagaaatcaatatgatcgtttctgacatcgaagctaacgccctctt                                                         <  1:765677‑M1/301‑58 (MQ=255)`  
                                                                                                                                                                                                                                                      `agaagaGTCACACTCATAGTCAACAGACTGGTCCAACGACACTGACTTCTGACAGGATTCTTGAcagttgtttcatatgaagagattgttaagtcacgataatcaataggagaaatcaatatgatcgtttctgacatcgaagctaacgccctcttagagagcgtcactaagttccactgcggggttatctacgactactccaccgctgagtacgtaagctaccgtccgagtgacttcggtgcgtatctggatgcgctggaagccgaggttgcacgaggcggtcttattgtgttccacaacg                                                       >  1:728584‑M1/1‑64 (MQ=255)`  
                                                                                                                                                                                                                                                      `agaagaGTCACACTCAGAGTCAACAGACTGGTCCAACGACACTGACTTCTGACAGGATTCTTGATGACTTTCCAGACGACTACGAGAAGTTTCGCTGGAGAGTCCCATTCTAATACGACTCACTAAAGGAGACACACCATGTTCAAACTGATTAAGAAGTTAGGCCAACTGCTGGTTCGTATGTACAACGTGGAAGCCAAGCGACTGAACGATGAGGCTCGTAAAGAGGCCACACAGTCACGataatcaataggagaaatcaatatgatcgtttctgacatcgaagctaacgccctcttag                                                       <  1:432217‑M1/301‑60 (MQ=255)`  
                                                                                                                                                                                                                                                        `aagaGTCACACTCAGAGTCAACAGACTGGTCCAACGACACTGACTTCTGACAGGATTCTTGATGACTTTCCAGACGACTACGAGAAGTTTCGCTGGAGAGTCCCATTCTAATACGACTCACTAAAGGAGACACACCATGTTCAAACTGATTAAGAAGTTAGGCCAACTGCTGGTTCGTATGTACAACGTGGAAGCAAAGCGACTGAACGATGAGGCTCGTAAAGAGGCCACACAGTCACGataatcaataggagaaatcaatatgatcttttctgacatcaaagctaacgccatcttagag                                                     <  1:737195‑M1/301‑62 (MQ=255)`  
                                                                                                                                                                                                                                                        `aagaGTCACACTCAGAGGCAACAGACTGGTCCAACGACACTGACTTCTGACAGGATTCTTGATGACTTGCCAGACGACTACGAGAAGTTTCGCTGGAGAGTCCCATTCTAATACGACTCACTAAAGGAGACACACCATGTTCAAACTGATTAAGAAGTTAGGCCAACTGCTGGTTCGTATGTACAACGTGGAAGCCAAGCGACTGAACGATGAGGCTCGTAAAGAGGCCACACAGTCACGataatcaataggagaaatcaatatgatcgtttctgacatcgaagctaacgccctcttagag                                                     <  1:18473‑M1/301‑62 (MQ=255)`  
                                                                                                                                                                                                                                                          `aggtcACACTCAGAGTCAACAGACTGGTCCAACGACACTGACTTCTGACAGGATTCTTGATGACTTTCCAGACGACTACGAGAAGTTTCGCTGGAGAGTCCCATTCTAATACGACTCACTAAAGGAGACACACCATGTTCAAACTGATTAAGAAGTTAGGCCAACTGCTGGTTCGTATGTACAACGTGGAAGCCAAGCGACTGAACGATGAGGCTCGTAAAGAGGCCACACAGTCACGataatcaataggagaaatcaatatgatcgtttctgacatcgaagctaacgccctcttagagag                                                   <  1:383424‑M1/299‑64 (MQ=255)`  
                                                                                                                                                                                                                                                            `gTCACACTCAGAGTCAACAGACTGGTCCAACGACACTGACTTC‑‑‑‑‑‑‑‑‑‑‑‑TGAcagttgtttcatatgaagagattgttaagtcacgataatcaataggagaaatcaatatgatcgtttctgacatcgaagctaacgccctcttagagagcgtcactaagttccactgctgggttatctacgactactccaccgctgagtacgtaagctaccgtcctagtgacttcggtgcgtatctggatgcgctggaagccgaggttgcacgacgcggtcttattgtgttccacaacggtcacaagtatgacgttc                                     >  1:8715‑M1/1‑46 (MQ=255)`  
                                                                                                                                                                                                                                                                     `agagTCAACAGACTGGTCCAACGACACTGACTTCTGACAGGTTTCTTGAcagttgtttcatatgaagagattgttaagtcacgataatcaataggagaaatcaatatgatcgtttctgacatcgaagctaacgccctcttagagagcgtcactaagttcaactgcggggttgtctacgactactccaccgctgagtacgtaagctaccgtccgagtgacttcggtgcgtatctggatgcgctggaagccgaggttgcacgaggaggtcttattgtgttccacaacggtcacaagtatgaca                                        <  1:596810‑M1/301‑253 (MQ=255)`  
                                                                                                                                                                                                                                                                         `tCAACAGACTGGTCCAACGACACTGACTTCTGACAGGATTCTTGAcagttgtttcatatgaagagattgttaagtcacgataatcaataggagaaatcaatatgatcgtttctgacatcgaagctaacgccctcttagagggagtcactaagttccactgcggggttatctacgactactccaccgctgagtacgtaagctaccgtccgagtgacttcggtgcgtatctggatgcgctggaagccgaggttgcacgaggcggtcttattgtgttccacaacggtcacaagtatgacgttcc                                    >  1:642604‑M1/1‑45 (MQ=255)`  
                                                                                                                                                                                                                                                                                  `tGGTCCAACGACACTGACTTCTGACAGGATTCTTGATGACTTTCCAGACGACTACGAGAAGTTTCGCTGGAGAGTCCCATTCTAATACGACTCACTAAAGGAGACACACCATGTTCAAACTGATTAAGAAGTTAGGCCAACTGCTGGTTCGTATGTACAACGTGGAAGCCAAGCGACTGAACGATGAGGCTCGTAAAGAGGCCACACAGTCACGataatcaataggagaaatcaatatgatcgtttctgacatcgaagctaacgccctcttagagagcgtcactaagttccactgcggggt                           <  1:370755‑M1/301‑88 (MQ=255)`  
                                                                                                                                                                                                                                                                                    `gTCCAACGACACTGACTTCTGACAGGATTCTTGAcagttgtttcatatgaagagattgttaagtcacgataatcaataggagaaatcaatatgatcgtttctgacatcgaagctaacgccctcttagagagcgtcactaagttccactgcggggttatctacgactactccaccgctgagtacgtaagctaccgtccgagtgacttcggtgcgtatctggatgagctggaagccgaggttgcacgaggcggtcttattgtgttccacaacggtcacaagctgtctcttatacacatctccg                         >  1:398830‑M1/1‑34 (MQ=255)`  
                                                                                                                                                                                                                                                                                         `aCGACACTGACTTCTGACAGGATTCTTGATGACGTTCCAGACGACTACGAGAAGTTTCGCTGGAGAGTCCCATTCTAATACGACTCACTAAAGGAGACACACCATGTTCAAACTGATTAAGAAGTTAGGCCAACTGCTGGTTCGTATGTACAACGTGGAAGCCAAGCGACTGAACGATGAGGCTCGTAAAGAGGCCACACAGTCACGataatcaataggagaaatcaatatgatcgtttctgacatcgaagctaacgccctcttagagagcgtcaataagttccactgcggggttatctac                    <  1:104786‑M1/301‑95 (MQ=255)`  
                                                                                                                                                                                                                                                                                            `acacTGACTTCTGACAGGATTCTTGATGACTTTCCAGACGACTACGAGAAGTTTCGCTGGAGAGTCCCATTCTAATACGACTCACTAAAGGAGACACACCATGTTCAAACTGATTAAGAAGTTAGGCCAACTGCTGGTTCGTATGTACAACGTGGAAGCCAAGCGACTGAACGATGAGGCTCGTAAAGAGGCCACACAGTCACGataatcaataggagaaatcaatatgatcgtttctgacatcgaagctaacgccctcttagatagcgtcactaagttccactgcggggttatctactgt                 >  1:31277‑M1/1‑204 (MQ=255)`  
                                                                                                                                                                                                                                                                                                           `aGGATTCTTGATGACTTTCCAGACGACTACGAGAAGTTTCGCTGGAGAGTCCCATTCTAATACGACTCACTAAAGTAGACACACCATGTTCAAACTGATTAAGAAGTTAGGCCAACTGCTGGTTCGTATGTACAACGTGGAAGCCAAGCGACTGAACGATGAGGCTCGTAAAGAGGCCACACAGTCACGataatcaataggagaaatcaatatgatcgtttctgacatcgaagctaacgccctcttagagagcgtcactaagttccactgcggggttatctacgactactccaccactgag  <  1:615160‑M1/301‑113 (MQ=255)`  

                                                                                                                                                                                                                                                                                                            `|`                                                                                                                                                                                                                                                                                                             
`CCCAGACAAAGGTAAAGCACATGAGGAAGGTCGCCCCGTTTCTATTACTGACCTACGTGGTTCTGGCGCACTACGCCAACTATCTGATACTATTATTGCCCTTGAGCGTAATCAGCAAGGCGATATGCCTAACCTTGTCCTCGTTCGTATTCTCAAGTGCCGCTTTACTGGTGATACTGGTATCGCTGGCTACATGGAATACAACAAGGAAACCGGATGGCTTGAACCATCAAGTTACTCAGGGGAAGAAGAGTCACACTCAGAGTCAACAGACTGGTCCAACGACACTGACTTCTGACAGGATTCTTGATGACTTTCCAGACGACTACGAGAAGTTTCGCTGGAGAGTCCCATTCTAATACGACTCACTAAAGGAGACACACCATGTTCAAACTGATTAAGAAGTTAGGCCAACTGCTGGTTCGTATGTACAACGTGGAAGCCAAGCGACTGAACGATGAGGCTCGTAAAGAGGCCACACAGTCACGCGCTCTGGCGATTCGCTCCAACGAACTGGCTGACAGTGCATCCACTAAAGTTACCGAGGCTGCCCGTGTGGCAAACCAAGCTCAACAGCTTTCCAAATTCTTTGAGTAATCA  >  NC_001604.1/12968‑13567`

  

Alignment Legend

Aligned base mismatch/match (shaded by quality score): `ATCG/ATCG` `< 3 ≤` `ATCG/ATCG` `< 15 ≤` `ATCG/ATCG` `< 27 ≤` `ATCG/ATCG` `< 37 ≤` `ATCG/ATCG` `< 38 ≤` `ATCG/ATCG`

Unaligned base: `atcg`    Masked matching base: `atcg`    Alignment gap: `‑`    Deleted base: `‑`

Reads not counted as support for junction

read\_name Not counted due to insufficient overlap past the breakpoint.

read\_name Not counted due to not crossing MOB target site duplication.
